# Acute Transverse Myelitis as an Uncommon Presentation of Neurobrucellosis: A Case Report

**DOI:** 10.1002/ccr3.70060

**Published:** 2025-01-02

**Authors:** Saja Karaja, Waddah Kazkz

**Affiliations:** ^1^ Faculty of Medicine Hama University Hama Syria

**Keywords:** brucella, brucellosis, case report, neurobrucellosis, transverse myelitis

## Abstract

Brucellosis mainly affects the musculoskeletal system with neurological manifestations observed in < 5% of all cases. This report outlines a unique case of neurobrucellosis that resulted in acute transverse myelitis (TM) with absent deep reflexes and negative Wright's reaction. In endemic regions, neurobrocellosis should be considered as a potential etiology of acute TM. Timely initiation of treatment is essential for optimizing clinical outcomes.

**Abbreviations**
APQ‐4: anti‐aquaporin‐4
BPH: benign prostatic hyperplasia
CNS: central nervous system
CRP: C‐reactive protein
CSF: cerebrospinal fluid
CT: computed tomography
EMG: electromyography
ESR: erythrocyte sedimentation rate
HTN: high blood pressure
IGg: immunoglobulin G
IV: intravenous
MOG autoantibodies: anti‐myelin oligodendrocyte glycoprotein
MRI: magnetic resonance imaging
PCR: polymerase chain reaction
TB: tuberculosis
TM: transverse myelitis
WBCs: white blood cells

## 1 Introduction

Brucellosis is a pervasive bacterial zoonotic ailment of global prevalence, characterized by a multifaceted systemic impact, encompassing a diverse array of clinical presentations that can manifest in acute, subacute, or chronic and relapsing iterations [1, 2, 3]. It is difficult to anticipate brucella infection in the absence of known prior exposure, as its diverse array of associated symptoms can closely resemble those of numerous other medical conditions [2, 4]. < 5% of brucellosis species can develop neurological symptoms [5]. Central nervous system (CNS) involvement is absolutely rare and when it happens it is usually associated with bad prognosis [3]. Neurobrucellosis usually manifests with some frequent presentations such as meningitis, encephalitis, meningovascular disease, or brain abscesses, while transverse myelitis (TM) is an unusual occurrence [3]. TM is an uncommon, acquired focal inflammatory disorder that equally affects both men and women of all ages [6]. It typically presents acutely so the term “transverse myelitis” often refers to either “transverse myelitis” or “acute transverse myelitis” [7]. TM often presents with common symptoms including motor and sensory deficits, in addition to autonomic dysfunction features [6]. Clinicians usually rely on the spinal cord magnetic resonance imaging (MRI) and cerebrospinal fluid (CSF) examination to diagnose myelitis [8]. Unfortunately, just one‐third of patients experience it with no lasting deficits [6]. In this report, we detail a unique case involving a 69‐year‐old patient who exhibited sudden onset symptoms of TM, characterized by paraplegia in the lower limbs, a distinct sensory level, and urinary incontinence, all of which developed rapidly over the course of a few hours. CSF analysis indicated a positive result for brucella polymerase chain reaction (PCR), while Wright's reaction yielded a negative outcome. It is atypical for neurobrucellosis to acutely precipitate transverse myelitis.

## 2 Case Presentation

### 2.1 Case History

A male patient, aged 69 years, was admitted to the hospital with the abrupt onset of bilateral paraplegia affecting the lower limbs, a clearly defined sensory level at the level of the first lumbar vertebrae (L1), and urinary incontinence, all developed within hours. Surgical history involved benign prostatic hyperplasia (BPH). The medical history involved an irregular intake of aspirin 81 mg and the treatment for hypertension with perindopril 5 mg, in addition to indapamide 12.5 mg intake. Clinical examination showed that the patient was oriented, cooperative, and conscious with normal vital signs, speech and fundus examination were normal, motor symptoms involved bilateral lower limbs paraplegia (0/5), while the upper limb power was normal (5/5). Sensory symptoms involved loss of all sensory modalities, paresthesia. All deep tendon and plantar reflexes were absent in both lower limbs and normal in the upper ones, with no cerebellar or extrapyramidal signs, and no cranial nerve involvement. Autonomic dysfunction leads to urinary incontinence, no pain, and no previous suffering from high‐grade fever in the last few months.

### 2.2 Differential Diagnosis, Investigations, and Treatment

**FIGURE 1:**
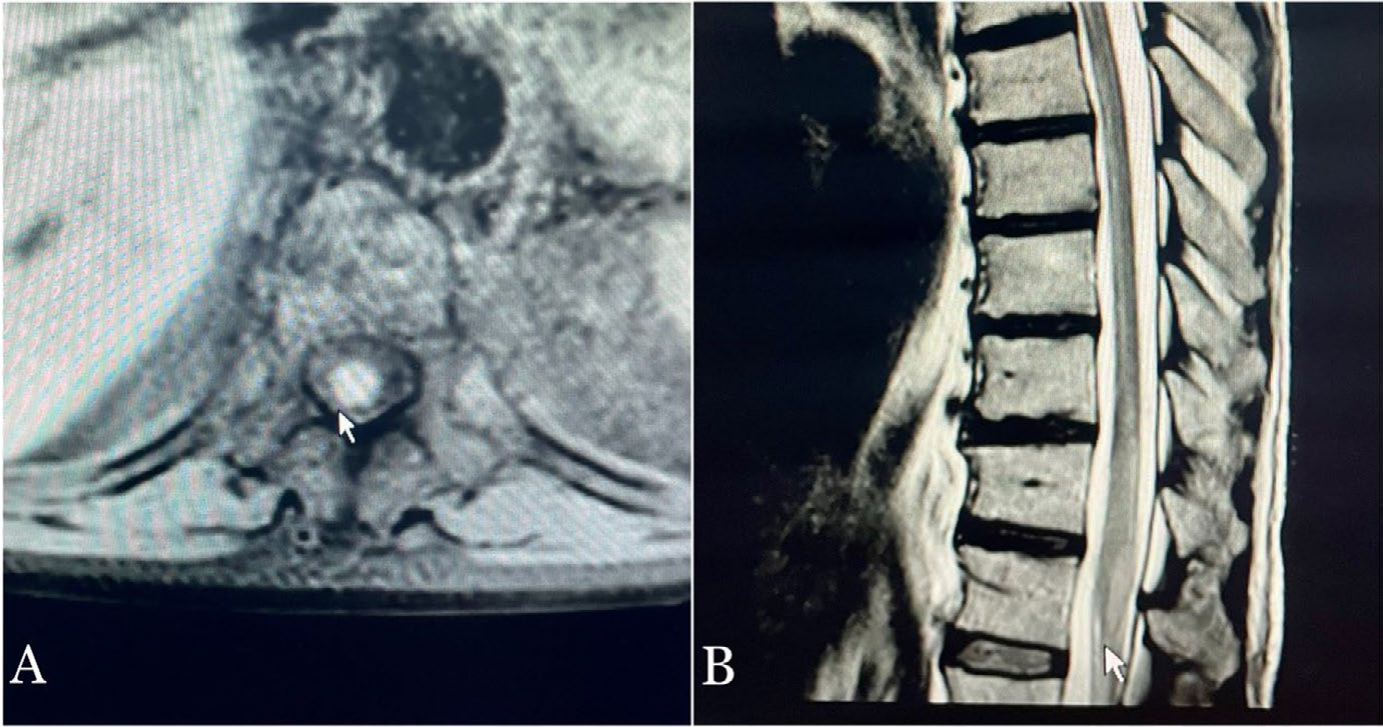
MRI of the entire spine with gadolinium contrast. (A) Axial T2‐weighted image at the level of T11 demonstrating a hyperintense signal within the spinal cord, suggestive of inflammation and edema. (B) Sagittal T2‐weighted image shows hyperintense signal changes at the level of T10–T12 thoracic vertebrae, indicative of an inflammatory process without compressive lesions. These findings are consistent with transverse myelitis.

Laboratory tests such as complete blood count revealed a white blood cells (WBCs) count of 26,900/μL, 90.9% of them were neutrophils. They also showed elevated inflammatory markers; C‐reactive protein (CRP) was 71.3, and later erythrocyte sedimentation rate (ESR) test was 60 mm/h. His other tests which included liver and renal function tests, electrolytes, and urine examination were all within normal limits. MRI study of the entire spine with gadolinium contrast provided evidence of T2 hyperintense signal changes at the level of (10‐11‐12) thoracic vertebrae, often related to an inflammatory process with no compressive lesion (Figure 1), so electromyography (EMG) was not performed. The patient was managed with short courses of intravenous (IV) antibiotic methylprednisolone solumedrol for 5 days, but he did not get better. During that time we continued with testing the CSF which was turbid with WBCs count of 30/μL, 95% of them are neutrophils with 2760 RBCs, glucose of 63 mg/dL, elevated levels of protein about 67 mg/dL, and immunoglobulin G (IGg) value in the CSF of 5.22 mg/dL with normal CSF IgG Index. There are no malignant cells detected in the examined CSF sample. Computed tomography (CT) of the chest excluded the tuberculosis (TB) infection and when (GeneXpert) PCR in the CSF was performed, it was negative. Immunology tests of anti‐aquaporin‐4 (APQ‐4)‐IgG autoantibodies and anti‐myelin oligodendrocyte glycoprotein (MOG) autoantibodies were negative. Electrophoresis showed positive oligoclonal bands correlated with systemic inflammation. The patient mentioned a history of unpasteurized dairy products so a real‐time PCR for brucella bacteria was performed and it was positive, while it is not usual for brucella bacteria to cause transverse myelitis. We were not able to identify the brucella type because that is not available in our country. The patient opted to transfer his care to another hospital, where the medical team considered the potential benefit of plasmapheresis. However, the intervention proved ineffective due to the advanced disease stage. At that time the patient was commenced on a 6‐week course of doxycycline 100 mg twice daily and rifampicin 600 mg once daily, alongside an intensive physical therapy program aimed at optimizing good outcomes.

### 2.3 Outcome and Follow‐Up

After a 6‐month course of physical therapy, the patient demonstrated improved test outcomes; however, enduring motor impairments persisted without any discernible recovery. Notwithstanding this, the patient began to experience sensations of pain and temperature.

## 3 Discussion

TM is an uncommon inflammatory neurological disorder. The designation “transverse” denotes the localization of the inflammatory process across the diameter of the spinal cord [7]. Our patient, aged 69 years, exemplifies the potential for TM to manifest at any stage of life, with increased prevalence documented among individuals in the vicinity of 10 and 20 years old, as well as those aged over 40 years [6, 7]. TM commonly presents with typical clinical features including motor deficits of rapid onset weakness, a variety of sensory symptoms such as pain, dysesthesia, and paresthesia at the affected level, as well as autonomic dysfunction features including urinary urgency, bladder or bowel incontinence, difficulty or inability to void, sexual dysfunction [6], and temperature dysregulation [7]. Around half of the patients experience complete paraplegia while all the patients exhibit a degree of bladder and bowel dysfunction [6]. Our patient presented at the hospital with bilateral rapid onset paraplegia of both lower limbs, clearly defined sensory level, and urinary incontinence, all of them developed within hours. A comparative analysis of the clinical manifestations observed in this case with those documented in previous reports of ATM attributed to brucellosis may provide valuable insights into the varied presentation and progression of neurobrucellosis‐associated TM. Clinical evaluation revealed bilaterally absent deep reflexes in the lower limbs. The location and extent of the lesion play a crucial role in determining the etiologic and predicting the outcome [8]. The primary cause of TM is often idiopathic, while the most commonly identified cause is demyelinating disease [6, 7]. Infectious myelitis can be caused by viral, bacterial, fungal, or parasitic etiologies [7]. The infection affects the spinal cord through a direct pathogenic effect and an immune‐mediated process, that often leads to clinical symptoms after a few weeks of the infection [8]. To diagnose TM, firstly we exclude a compressive cord lesion with an MRI study of the entire spine that confirmed the inflammation by providing an evidence of T2 hyperintense signal changes at the level of T (10‐11‐12), laboratory tests revealed a high level of WBCs and CSF analysis showed pleocytosis, in addition to the features the patient came with, they were enough to diagnose TM [6]. We thought of infectious reasons because the symptoms developed within hours [8]. The patient lives in TB endemic area, so neurotuberculosis was excluded by performing the chest CT and when the result of (GeneXpert) PCR in the CSF was negative, the protein in the CSF was high but not that much, a very high CSF protein level is among the known features of neurotuberculosis [9]. Glucose in the CSF was within accepted limits while low CSF glucose concentration generally suggests fungal, bacterial, or mycobacterial infection, especially when it is associated with an elevated CSF white blood cell count [7], pleocytosis with neutrophils are predominant suggests a bacterial or mycobacterial infection [7]. Real‐time PCR for brucella was performed because the patient has a history of unpasteurized dairy products and it was positive. Positive brucella PCR in any clinical specimen is a confirmed infection [10]. Brucella is a small, gram‐negative coccobacillus [2]. It causes brucellosis, also known as malta fever which is a highly prevalent zoonotic disease worldwide [9]. It remains endemic in the Middle East, Mediterranean Europe, Africa [9], and Latin America [5]. Brucella can survive for a long time on various surfaces in the environment. It can then enter the body through the respiratory, digestive system, eyes, or even damaged skin [1], but it is mainly transmitted to humans through direct contact with infected animals or consumption of their contaminated products [2]. *Brucella abortus* , *B. melitensis* , *B. canis* , and *B. suis* are the main four species of brucella [5]. Our case reports a patient with brucella presents with unusual symptoms of pyrexia, asthenia, muscle arthralgia, chills, or even hyperhidrosis [1] because it did not affect the musculoskeletal system as it mainly does. In our case, human brucellosis affects the CNS, while < 5% of all brucella species develop neurologic symptoms [5]. Besides being rare, CNS involvement is often associated with a bad prognosis [3], and it usually manifests with meningitis, radiculitis, encephalitis, meningovascular disease, brain abscesses, or demyelinating syndromes, while transverse myelitis is an unusual occurrence, and is reported in only 1.2%–3% of all neurobrucellosis cases [3]. These manifestations usually result from the direct invasion of the tissues [11]. Myelitis is usually classified as a chronic CNS involvement of neurobrucellosis [9] but in our case the symptoms developed within hours. Common symptoms of neurobrucellosis are fever, headache, nausea, and vomiting [9], our patient presented with none of these previous symptoms but a rapid onset of lower limbs paraplegia, clearly defined sensory level at the level L1 and urinary incontinence which are common TM symptoms. Treating neurobrucellosis with antibiotics remains a significant challenge primarily attributable to the intracellular localization of the bacteria [12]. The most effective pharmaceutical drugs for the treatment of brucellosis are tetracyclines, sulphonamides, streptomycin, and rifampicin [12]. A later study suggested starting treating neurobrucellosis with a combination of intravenous ceftriaxone with oral therapy [13]. Before neurobrucellosis diagnosis, the patient was managed with short courses of IV antibiotic methylprednisolone and solumedrol for 5 days to treat the inflammation but he did not get better and when we diagnosed the neurobrucellosis the patient preferred to transfer his medical care to an alternate healthcare facility where plasmapheresis was considered as a potential treatment modality. Regrettably, due to the advanced disease progression, plasmapheresis did not yield the desired clinical improvement. At that time the patient was put on a 6‐week antibiotic course comprising doxycycline 100 mg twice daily and rifampicin 600 mg administered once daily with an intensive physical therapy to optimize treatment outcomes. After 6 months of physical therapy patient's lower limb power is still as it was 0/5, and now he can feel the pain and heat, with better laboratory test results. Recovery outcomes vary, with one‐third of patients with TM fully recovering, one‐third with a moderate level of permanent disability, and the last one‐third with lasting deficits [6]. This report would benefit from further discussion of diagnostic strategies and treatment approaches to offer comprehensive guidance for managing this condition.

## 4 Conclusion

It is vital to recognize neurobrucellosis as a conceivable cause of rapidly progressive transverse myelitis, especially in populations residing in endemic regions. Moreover, it is essential to emphasize the likelihood of neurobrucellosis presenting with uncharacteristic symptoms, potentially leading to misdiagnosis. Wright's reaction may not consistently provide accurate diagnostic results for neurobrucellosis and has the potential to yield negative outcomes in certain instances of brucellosis. Prompt initiation of therapeutic interventions is crucial, as delays in commencing treatment may increase the risk of enduring permanent complications.

## Author Contributions

**Saja Karaja:** data curation, writing – original draft, writing – review and editing. **Waddah Kazkz:** supervision.

## Ethics Statement

The authors have nothing to report.

## Consent

Written informed consent was obtained from the patient for publication of this case report. A copy of the written consent is available for review by the Editor‐in‐Chief of this journal.

## Conflicts of Interest

The authors declare no conflicts of interest.

## Data Availability

No datasets were generated or analyzed during the current study.
